# Leisure-Time Physical Activity and All-Cause Mortality

**DOI:** 10.1371/journal.pone.0101548

**Published:** 2014-07-02

**Authors:** Jouni Lahti, Ansku Holstila, Eero Lahelma, Ossi Rahkonen

**Affiliations:** Hjelt Institute, Department of Public Health, University of Helsinki, Helsinki, Finland; TaipeiCityHospital, Taiwan

## Abstract

**Background:**

Physical inactivity is a major public health problem associated with increased mortality risk. It is, however, poorly understood whether vigorous physical activity is more beneficial for reducing mortality risk than activities of lower intensity. The aim of this study was to examine associations of the intensity and volume of leisure-time physical activity with all-cause mortality among middle-aged women and men while considering sociodemographic and health related factors as covariates.

**Methods:**

Questionnaire survey data collected in 2000-02 among 40–60-year-old employees of the City of Helsinki (N = 8960) were linked with register data on mortality (74% gave permission to the linkage) providing a mean follow-up time of 12-years. The analysis included 6429 respondents (79% women). The participants were classified into three groups according to intensity of physical activity: low moderate, high moderate and vigorous. The volume of physical activity was classified into three groups according to tertiles. Cox regression analysis was used to calculate hazard ratios (HR) and 95% confidence intervals (CIs) for all-cause mortality.

**Results:**

During the follow up 205 participants died. Leisure-time physical activity was associated with reduced risk of mortality. After adjusting for covariates the vigorous group (HR = 0.54, 95% CI 0.34–0.86) showed a reduced risk of mortality compared with the low moderate group whereas for the high moderate group the reductions in mortality risk (HR = 0.72, 95% CI 0.48–1.08) were less clear. Adjusting for the volume of physical activity did not affect the point estimates. Higher volume of leisure-time physical activity was also associated with reduced mortality risk; however, adjusting for the covariates and the intensity of physical activity explained the differences.

**Conclusions:**

For healthy middle-aged women and men who engage in some physical activity vigorous exercise may provide further health benefits preventing premature deaths.

## Introduction

There is an extensive amount of studies showing that physical activity is important to health benefits and reducing mortality risk [1]. Some studies suggest that avoiding physical inactivity is the most important [2], [3] and that a higher volume of physical activity further reduces mortality risk indicating a dose-response association [3]. In addition, a recent review and meta-analysis suggested a dose-response association between non-vigorous physical activity and mortality [4]. Another recent review and meta-analysis showed that vigorous intensity physical activity is associated with even larger reductions in mortality risk than activities of lower intensity [5]. However, these findings may be related to the higher volume of activity and not the intensity per se. Thus, it is still poorly understood whether vigorous intensity physical activity is more beneficial for reducing mortality risk than activities of lower intensity [5].

As those who engage in vigorous physical activity may have a higher volume of physical activity and thus lower the risk of mortality, the volume of physical activity needs to be considered in the analyses to separate the independent contribution of vigorous activity on reduced mortality risk. In addition, in epidemiological studies examining physical activity and mortality, existing physical as well as mental health differences need to be considered in the analysis because health problems potentially affect physical activity [6]. Furthermore, it is also important to take into account other covariates that are risk factors for mortality and may be unequally distributed between physical activity groups [7], for example socioeconomic position [8], [9], BMI, smoking and drinking problems [8], [10].

The aim of this study was to examine the associations of the intensity and volume of leisure-time physical activity with all-cause mortality among middle-aged women and men while considering sociodemographic and health related factors as covariates. Additional aim was to examine the independent association of the intensity of leisure-time physical activity by adjusting for the volume of leisure-time physical activity in the analyses and vice versa for the volume of leisure-time physical activity.

## Materials and Methods

### Ethics statement

The study is approved by the ethics committees of the Department of Public Health, University of Helsinki and the health authorities of the City of Helsinki. Informed, written consent was obtained from all participants in the study.

### Data sources

Helsinki Health Study baseline questionnaire surveys were conducted in 2000, 2001 and 2002 among 40- to 60-year-old employees of the City of Helsinki. The sample consisted of 13 346 persons and 7148 women and 1799 men returned the questionnaire (response rate 67%). Younger employees, men and manual workers were somewhat underrepresented among the respondents, but otherwise the data satisfactorily represent the target population [11], [12].

Mortality data were obtained from the registers of Statistics Finland. All deaths until the end of year 2012 were individually linked with the questionnaire data (74% gave permission to the linkages), providing a mean follow-up time of 12-years. There was missing information in some study variables (n = 149). Those, reporting no leisure-time physical activity (n = 28) were excluded from the analyses. This yielded 5051 women and 1378 men for the analyses.

### Leisure-time physical activity

The respondents were asked their average weekly hours of leisure-time physical activity/exercise (including commuting) within the previous 12 months. The respondents were instructed first to estimate the intensity of each activity they engaged in and then to estimate how many hours per week they spent on average in physical activity corresponding to each grade of intensity. Four grades of intensity were given exemplified by common activities: walking, brisk walking, jogging, and running, or their equivalent activities. Each intensity grade had five response alternatives: no activity, 0–½ hours/week, ½–1 hours/week, 2–3 hours/week, ≥4 hours/week. We classified the participants into three groups according to the highest intensity of physical activity they engaged in: low moderate (e.g. walking), high moderate (e.g. brisk walking), and vigorous (e.g. jogging/running). The volume of physical activity was assessed by approximate metabolic equivalents (MET) [13]. MET-hours per week were calculated by multiplying the time used (weekly hours) by the estimated MET value of each physical activity grade [14] ^,^ and then adding the four values together. The volume of leisure-time physical activity was classified into three groups according to tertiles (cutpoints: 14.75 and 34.75 MET-hours/wk) [15].

### Covariates

Age included five groups at baseline: 40, 45, 50, 55 and 60 years. Smoking was categorized into non-smokers, ex-smokers and smokers. Drinking problems were measured by the CAGE questionnaire [16]. Four occupational social classes were used as an indicator of socioeconomic position (SEP): managers (managerial and administrative work) and professionals (e.g. teachers and doctors), semi-professionals (e.g. nurses and foremen), routine non-manual employees (e.g. child minders and assistant maids), and manual workers (e.g. transport and cleaning work) [17]. The respondents were asked how strenuous their work is physically and mentally. Physical strenuousness of work was dichotomized as heavy or light. The same procedure was repeated for mental strenuousness of work. Body mass index (BMI) was calculated, using questionnaire data on height (m) and weight (kg). Physical functioning was measured by the physical functioning sub-scale of the Short-Form 36 (SF-36) health questionnaire [18]. Mental health was measured by the general mental health sub-scale of the SF-36. These SF-36 scores range from zero to 100, higher scores indicate better health, and mean scores between 45–54 years are 84.6 and 75.3 for physical functioning and mental health, respectively [18]. Details of limiting longstanding illness (LLI) were requested in two separate questions and classified into two groups: those with and those without LLI.

### Statistical methods

First descriptive statistics were calculated. Cox regression analysis was used to calculate hazard ratios (HR). In model 1 age and gender were adjusted for. In model 2 additional adjustments were made for smoking, drinking problems, occupational social class, physical and mental strenuousness of work. In model 3 additional adjustments were made for BMI, physical functioning, mental health and LLI. In model 4 the HRs were further adjusted for the volume or the intensity of leisure-time physical activity. The proportional hazards assumption was examined using Schoenfeld residuals [19] and confirmed for leisure-time physical activity and covariates. SPSS 20.0 statistical package was used.

## Results

Table 1 presents the distributions of baseline study variables (Table 1). Increasing intensity and volume both showed younger age. Women were less often vigorously active than men but in the volume groups gender differences were smaller. Increasing intensity and volume also showed less smoking but differences in drinking problems were minor and somewhat inconsistent. Increasing intensity was associated with higher SEP but between the volume groups there were smaller SEP differences. There were some differences in the physical and mental strenuousness of work. Increasing intensity as well as volume was associated with lower BMI, better physical functioning, mental health and lower prevalence of LLI. Physical activity intensity and volume were inter-related. Over half of the vigorously active was in the highest third of physical activity volume whereas less than every tenth was in the lowest third. Of the high moderate group, the pattern was different; less than a quarter was in the highest third and over forty percent in the lowest third. Of the low moderate group, almost eighty percent were in the lowest third whereas none were in the highest third.

**Table 1:** Description of study variables by leisure-time physical activity.

	Leisure-time physical activity							
	Intensity				Volume			
	Low moderate	High moderate	Vigorous	p*	Low	Middle	High	p*
**N**	532	3543	2354		2146	2076	2207	
**Age in years (mean)**	51.3	50.0	48.0	<0.001	50.1	49.3	48.8	<0.001
**Women (%)**	77.8	83.7	71.1	<0.001	79.0	80.1	76.7	0.019
**Smokers (%)**	30.3	24.5	18.8	<0.001	26.5	22.6	19.7	<0.001
**Ex-smokers (%)**	22.2	24.2	23.7		23.1	23.8	24.6	
**Drinking problems (%)**	16.9	16.9	18.7	0.163	19.8	17.0	15.9	0.002
**SEP** 1 **(%)**				<0.001				0.049
Manual workers	18.2	14.5	12.4		14.8	12.2	15.0	
Routine non-manuals	38.9	37.5	29.1		34.3	35.5	33.8	
Semi-professionals	16.2	19.2	20.8		18.3	20.0	20.4	
Managers/professionals	26.7	28.8	37.7		32.7	32.3	30.8	
**Physically strenuous work (%)**	33.5	35.1	28.8	<0.001	30.5	33.2	34.2	0.028
**Mentally strenuous work (%)**	18.0	14.3	13.3	0.018	15.8	14.3	12.2	0.003
**BMI** 2 **(mean)**	27.7	26.1	24.4	<0.001	26.8	25.3	24.7	<0.001
**Physical functioning (mean)**	78.3	88.1	93.7	<0.001	85.5	90.1	92.4	<0.001
**Mental health (mean)**	75.1	78.2	80.2	<0.001	76.4	78.5	81.0	<0.001
**LLI** 3 **(%)**	27.6	19.7	11.1	<0.001	22.0	15.6	14.0	<0.001
**Volume/intensity (%)**				<0.001				<0.001
Low/Low moderate	77.8	43.6	7.9		19.3	5.7	0.0	
Middle/Low moderate	22.2	33.1	33.4		72.0	56.5	37.4	
High/High moderate	0.0	23.3	58.7		8.7	37.9	62.6	

1 socioeconomic position,

2 body mass index (kg/m^2^),

3 limiting longstanding illness.

*χ2/ANOVA.

During the follow up 205 (2.6% of women and 5.2% of men) participants died (Table 2). According to the intensity of leisure-time physical activity, those engaging in low moderate-intensity activities (6.0%) had the highest mortality rate and those engaging in vigorous activities (2.2%) had the lowest rate. For the high moderate-intensity group the mortality rate was 3.4%. According to the volume of leisure-time physical activity, the lowest third had the highest mortality rate (4.3%), whereas for the highest (2.7%) and middle thirds (2.5%) the mortality rates were somewhat lower. More than half of all deaths were cancer related (ICD10 codes C00-D48). Among women breast cancer was the most common cancer causing death whereas among men the most common cancer was lung cancer. Cardiovascular disease (ICD-10 codes I00-I99) was the second common diagnostic group causing one fourth of the deaths. Among both women and men the most common cardiovascular disease was arteriosclerotic vascular disease.

**Table 2:** All-cause mortality (number of deaths (%)) during the follow-up by physical activity intensity and volume groups.

Physical activity	All			Women			Men		
	n		p*	n		p*	n		p*
**Intensity**	6429	205 (3.2)	<0.001	5051	133 (2.6)	0.01	1378	72 (5.2)	<0.001
Low moderate	532	32 (6.0)		414	19 (4.6)		118	13 (11.0)	
High moderate	3543	122 (3.4)		2964	81 (2.7)		579	41 (7.1)	
Vigorous	2354	51 (2.2)		1673	33 (2.0)		681	18 (2.6)	
**Volume**	6429	205 (3.2)	0.001	5051	133 (2.6)	0.061	1378	72 (5.2)	0.005
Low	2146	93 (4.3)		1696	57 (3.4)		450	36 (8.0)	
Middle	2076	52 (2.5)		1663	35 (2.1)		413	17 (4.1)	
High	2207	60 (2.7)		1692	41 (2.4)		515	19 (3.7)	

*χ2.

Firstly, we examined the association of the three intensity grades of leisure-time physical activity with mortality adjusting for covariates and the volume of leisure-time physical activity and using Cox regression analyses. The associations between the intensity of leisure-time physical activity and mortality were similar for women and men (p = 0.2 for interaction) and women and men were pooled for the analyses. Compared with the low moderate group, the vigorous (HR = 0.47, 95% CI 0.30–0.73) group had clearly reduced mortality risk when age, gender and other confounders were adjusted for in model 2 (Table 3). Also the high moderate group had reduced mortality risk (HR = 0.66, 95% CI 0.45–0.98). When additional adjustments were made for BMI, physical functioning, mental health and LLI the associations attenuated and remained for the vigorous group (HR = 0.54, 95% Cl 0.34–0.86) but lost statistical significance for the high moderate group. Adjusting for the volume of leisure-time physical activity had no effects on the point estimates.

**Table 3:** Hazard ratios (95% CI) for all-cause mortality according to the intensity of leisure-time physical activity.

HR (95%CI) according to the intensity of physical activity			
	Low moderate	High moderate	Vigorous
Model 1	1.00	0.64 (0.44–0.95)	0.43 (0.27–0.67)
Model 2	1.00	0.66 (0.45–0.98)	0.47 (0.30–0.73)
Model 3	1.00	0.72 (0.48–1.08)	0.54 (0.34–0.86)
Model 4	1.00	0.73 (0.49–1.11)	0.55 (0.32–0.94)

Model 1 adjusted for age and gender.

Model 2 adjusted for covariates in model 1 and additionally for occupational social class, physical and mental strenuousness of work, smoking and drinking problems.

Model 3 adjusted for covariates in model 2 and additionally for body mass index, physical functioning mental health and limiting longstanding illness.

Model 4 adjusted for covariates in model 3 and additionally for the volume of physical activity.

We then examined the contribution of the volume of leisure-time physical activity classified into three groups according to tertiles. The associations were similar for women and men (p = 0.5 for interaction) and women and men were pooled for the analyses. Compared with the lowest third, the middle (HR = 0.61, 95% CI 0.44–0.86) and the highest (HR = 0.67, 95% CI 0.48–0.93) thirds had reduced mortality risk when age and gender were adjusted for (Table 4). When additional adjustments were made for other confounders in model 2, the association lost statistical significance for the highest third. Further adjustments for BMI, physical functioning, mental health and LLI attenuated the associations and statistical significance was lost for both the middle (HR = 0.71, 95% CI 0.50–1.00) and the highest (HR = 0.81, 95% CI 0.58–1.14) thirds. Adjusting for the intensity of leisure-time physical activity further attenuated the associations and no clear patterns remained.

**Table 4:** Hazard ratios (95% CI) for all-cause mortality according to the volume of leisure-time physical activity.

HR (95%CI) according to the volume of physical activity			
	Low	Middle	High
Model 1	1.00	0.61 (0.44–0.86)	0.67 (0.48–0.93)
Model 2	1.00	0.65 (0.47–0.92)	0.73 (0.53–1.01)
Model 3	1.00	0.71 (0.50–1.00)	0.81 (0.58–1.14)
Model 4	1.00	0.78 (0.55–1.12)	0.99 (0.67–1.47)

Model 1 adjusted for age and gender.

Model 2 adjusted for covariates in model 1 and additionally for occupational social class, physical and mental strenuousness of work, smoking and drinking problems.

Model 3 adjusted for covariates in model 2 and additionally for body mass index, physical functioning mental health and limiting longstanding illness.

Model 4 adjusted for covariates in model 3 and additionally for the intensity of physical activity.

## Discussion

We aimed to examine whether the intensity and volume of leisure-time physical activity were associated with all-cause mortality among middle-aged employees after considering key covariates. Increasing intensity and volume of leisure-time physical activity was associated with reduced mortality risk similarly among women and men. Those engaging in vigorous activity had clearly reduced mortality risk compared with those engaging in low moderate-intensity activity, whereas, high moderate-intensity activity such as brisk walking showed weaker associations. The volume of leisure-time physical activity did not explain the reduced risk found for the vigorous intensity physical activity. The higher volume of leisure-time physical activity was also associated with reduced mortality risk; however, adjusting for the covariates and the intensity of physical activity explained the differences.

The findings of this study suggest that vigorous activity in particular is associated with reduced mortality independently of the volume of leisure-time physical activity and other covariates. Few previous studies have examined the intensity of physical activity controlling for the volume of physical activity using different methods and health outcomes [15], [20]–[23]. In general, the results of these studies are in accordance with our study. Only one previous study has examined the intensity of physical activity and mortality [20]. The study by Lee and Paffenbarger was conducted only among men and showed that vigorous physical activity was clearly associated with reduced mortality risk and moderate-intensity activity showed some, albeit less clear reductions in mortality risk [20]. Another study among men showed that vigorous physical activity reduced the risk of coronary heart disease but moderate-intensity activity showed weaker associations [21]. A cross sectional study using objective physical activity measurement showed that vigorous physical activity was associated with reduced risk of metabolic syndrome independently of the physical activity volume among women and men [23]. Also our previous studies have showed that vigorous activity is more beneficial than moderate-intensity physical activity for various domains of health including sickness absence [15] and physical health functioning [22].

Previous studies have suggested a dose-response association between physical activity volume and mortality [3]. Some studies, however, suggest that higher amounts of activity do not further reduce mortality risk [2] which is in accordance with our results. However, in our study the association of the volume of leisure-time physical activity with mortality was not as strong as for the intensity, and the association was explained by the intensity of physical activity and other covariates. It should be noted that in addition to the intensity and volume also the type of physical activity may be important for health benefits and reduced mortality [2], [24].

There are some methodological issues when examining the independent associations of the intensity and volume of physical activity with health outcomes. The intensity and volume of physical activity are inter-related as the volume (MET-hours/wk) is the product of intensity and time spent in physical activity. Furthermore, those who engage in vigorous physical activity are also likely to engage in lower intensity activity. In these data, less than five per cent of the vigorously active did not engage in high or low moderate activities (data not shown). There are different methods, described in previous studies [15], [20]–[23], to examine the associations of different intensity physical activity with various health outcomes, independent of the volume of physical activity. We used multivariate models similar to study by Tanasescu et al. [21]. A statistical problem may, however, arise in multivariate models if highly correlated (correlation coefficient>0.60) variables are in the same model i.e. multicollinearity. When we examined the intensity of physical activity, adjusting for the volume of physical activity slightly widened the confidence intervals which may indicate multicollinearity. However, the correlation between the intensity and volume of physical activity was moderate (correlation coefficient = 0.52) and thus should not pose a statistical problem in the analyses.

When examining physical activity and mortality taking account of baseline health is important as existing health problems may restrict physical activity in general and, vigorous activity in particular. This has been somewhat ignored in previous studies [20]. We adjusted extensively for baseline health related covariates including BMI, LLI, physical functioning and mental health. For example, the SF-36 physical functioning subscale indicates health problems that affect physical functioning such as participation in physical activities. These factors may be partial mediating factors between leisure-time physical activity and mortality and there is a risk of overadjustment. Our sensitivity analyses showed that of the covariates included, physical functioning attenuated the examined associations the most but the attenuating effect was relative small and the associations remained strong for the vigorous group even after all adjustments.

Regarding the highest intensity of leisure-time physical activity less than every tenth reported only low moderate-intensity physical activity such as walking. About half reported high moderate-intensity activity such as brisk walking and more than every third vigorous activity such as jogging or running. Men engaged in vigorous activity more often than women and consequently a large part of women engaged in high moderate-intensity activity. In addition, the cutpoint i.e. 14.75 MET-hours per week for the lowest third of physical activity volume is comparable to 2.5 hours of brisk walking per week (15 MET-hours) which is the base of physical activity recommendations [1]. Thus the studied cohort is relatively active physically compared with European adults in general [25]. Also vigorous activity was quite common and thus achievable for many middle-aged.

Men had a higher mortality rate compared with women which is in accordance with the general middle-aged population in Finland [26]. Among middle-aged Finns the most common cause of death is still coronary heart disease, although cardiovascular disease mortality in general has decreased. Also cancers (e.g. lung cancer among men and breast cancer among women) are common causes of death. Other common causes include alcohol related deaths and suicides [26]. In this study, the relatively low number of deaths during the follow-up did not allow detailed cause-specific analyses of mortality. However, the most common diagnostic group was cancer accounting for half of the deaths and the second common diagnostic group was cardiovascular disease accounting for one fourth of the deaths. Our sensitivity analyses showed similar patterns between leisure-time physical activity and cancer mortality as with all-cause mortality (data not shown).

A number of biological mechanisms for the association between physical activity and mortality have been proposed in previous studies [5]. Biological mechanisms proposed include e.g. increase in the high-density lipoproteins and reduction of coronary artery calcium, however, these mechanisms relate cardiovascular diseases and since in these data over half of the deaths were cancer related other mechanism needs to be considered. It has been proposed that exercise may improve anti-tumor immune defenses and antioxidant defense systems [27]. Also other mechanisms have been proposed such as reduced abdominal fat, however, in these data adjusting for BMI had only minimal effects on the association between physical activity and mortality. On the other hand, BMI does not directly measure abdominal fat. In addition, there is evidence that vigorous activity reduces the risk of colorectal cancer more than activities of lower intensity [28], however, the intensity of physical activity is not related to the risk of breast cancer although there is a dose-response association between physical activity volume and breast cancer [29]. As higher intensity of physical activity contributes to fitness more than activities of lower intensity the potential mechanisms regarding the intensity are probably at least partly related to fitness. Previous studies have shown that increased fitness at middle-age reduce the risk of mortality, comparable to smoking cessation [30]. Nonetheless, as the scientific evidence for the potential mechanisms is limited, further studies are needed.

### Strengths and limitations

We were able to take into account key health related covariates, including LLI, physical functioning and mental health which is highly important as those with health problems may be unable to engage in physical activity, especially, with higher intensity. There were only few people that did not engage in any leisure-time physical activity thus comparison with them was not possible. This study focuses on leisure-time physical activity and other activities such as household activities that contribute to energy expenditure were not considered in the analyses, which is a limitation. In addition, other aspects of leisure-time physical activity such as the type of activity were not examined. Leisure-time physical activity was self-reported and lacks formal validation. However, other questionnaire data as well as objectively measured physical activity [23] have produced similar findings and no single questionnaire has proven superior for measuring leisure-time physical activity in large surveys [31]. The use of objectively measured physical activity would have added to the study, however, in large datasets questionnaires are still the first choice of measurement. There are characteristics in our data that limit the generalizability of the findings. The participants were middle-aged municipal employees and 80% of them were women which corresponds to the gender distribution of the municipal sector in general and the staff of the City of Helsinki. We pooled women and men in the analyses and thus these results are female dominated. However, the association between physical activity and mortality was not statistically significantly different between women and men.

### Conclusions

Increasing intensity and volume of leisure-time physical activity was associated with reduced mortality risk. Vigorous activity was clearly associated with reduced mortality risk independently of the volume of leisure-time physical activity. For healthy middle-aged women and men who engage in some physical activity vigorous exercise may provide further health benefits preventing premature death. Future studies examining physical activity and mortality risk should consider the intensity of physical activity more carefully.
